# Electro-Magnetism

**Published:** 1860-10

**Authors:** 


### ELECTRO-MAGNETISM.

This agency for the removal of various ailments is of doubtful value; if it had any uniform efficiency, it would long before now have become an accredited means for the cure of disease among educated physicians; it has not become so, chiefly because of its uncertain and transient effects, the costliness of its application, and the need of its prolonged employment. It ought to be a sufficient ground for its being regarded with disfavor, that it is almost entirely practiced by ignorant persons, foreigners, and peripatetics; that its application has been succeeded sometimes by an abatement or removal, more or less permanent, of the symptoms, is not disputed, but these good results have not followed with sufficient frequency to enable unprejudiced and intelligent minds to feel satisfied that electro-magnetism was the cause of the cure, while the immense multitude of perfect failures, to say nothing of disastrous succeedences, have operated to produce the general impression on the minds of educated physicians of various "schools," that up to this time, electro-magnetism, as applied to the removal of disease, is among the quackeries of the age.

